# Reusable EGaIn-Injected Substrate-Integrated-Waveguide Resonator for Wireless Sensor Applications

**DOI:** 10.3390/s151128563

**Published:** 2015-11-11

**Authors:** Muhammad Usman Memon, Sungjoon Lim

**Affiliations:** School of Electrical and Electronics Engineering, Chung-Ang University, 84 Heukseok-ro, Dongjak-gu, Seoul 156-756, Korea; E-Mail: musmanm@outlook.com

**Keywords:** wireless sensors, multi-resonator, SIW, EGaIn, liquid metal, RFID, chipless tag, barcode

## Abstract

The proposed structure in this research is constructed on substrate integrated waveguide (SIW) technology and has a mechanism that produces 16 different and distinct resonant frequencies between 2.45 and 3.05 GHz by perturbing a fundamental *TE*_10_ mode. It is a unique method for producing multiple resonances in a radio frequency planar structure without any extra circuitry or passive elements is developed. The proposed SIW structure has four vertical fluidic holes (channels); injecting eutectic gallium indium (EGaIn), also known commonly as liquid metal (LM), into these vertical channels produces different resonant frequencies. Either a channel is empty, or it is filled with LM. In total, the combination of different frequencies produced from four vertical channels is 16.

## 1. Introduction

The most promising wireless sensor application is the radio frequency identification (RFID), that is a technology based on the mutual exchange of information through radio signal propagation [1]. Owing to its quality of identifying and tracking items, RFID systems are used for many applications: healthcare, facility management, aviation, security, retailing, *etc*. In the retailing or food chain business, it is very important to keep track of the localization and the history of items to increase quality of service and security in the food chain. In [2], the authors discussed the application of RFID sensors to cold chain logistics of fresh products. Mennecke and Townsend [3] proposed an RFID system to determine the product provenance in the meat production industry. Furthermore, in consumer packaged goods management, several important companies require their providers to install RFID tags to pallets or boxes to improve the processes of storage, inventory, and security. In the field of healthcare, RFID technologies are used to manage different aspects of a hospital. Some tasks, such as blood transfusions, are controlled by means of RFID technologies [4] to find the correct blood bag for a specific patient. Amini *et al.* [5] proposed the use of an RFID system to collect data related to the movement of trauma patients. Tracking the position of components in a manufacturing chain is commonly carried out by means of RFID technologies. For instance, in robot construction [6], the integration of RFID devices in the fabrication process allows information to be collected about upcoming tasks. Infineon Technologies, one of the largest semiconductor manufacturers in the world, has created an identification and localization system using RFID and ultrasound sensors to improve the logistics in the wafer fabrication process [7].

**Figure 1:**
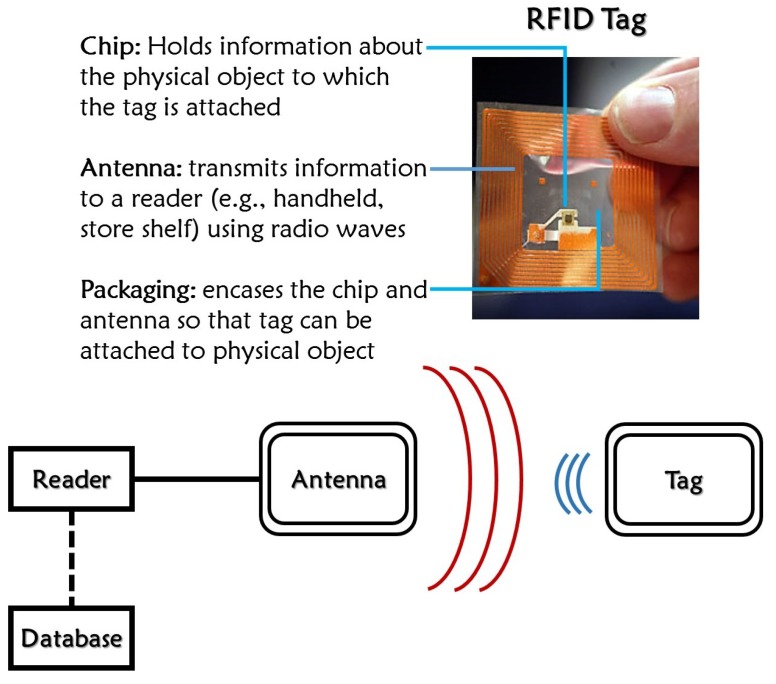
Wireless sensor scenario (radio frequency identification (RFID) system).

RFID systems have emerged from one of the branches of auto-ID systems that utilizes RF (radio waves) to identify objects. Currently, there is a huge interest in the development and research of different RFID systems to replace conventional barcode systems. Although barcodes have a low price in the market, they still have many drawbacks, including the need for a line-of-sight to read, sensitivity to wear and tear, and risk of tampering. With the help of RFID technology, these difficulties can easily be resolved. However, cost effectiveness remains as a concerning issue. RFID systems usually include two prominent parts: the tag (transponder) and the reader (interrogator) as shown in Figure 1. The reader and tag use an RF interface for communication between them. There are generally three kinds of RFID tags available: active (includes an onboard battery and works like a beacon), semi-active (includes an onboard battery but needs signal energy from the reader to transmit the interrogation signal), and passive (totally relies on the signal energy of the RFID reader to communicate). Of these kinds of tags, passive tags are the cheapest; of those with a passive nature, the chipless tags are at the front line in research for low-cost tags.

RFID systems have few dedicated industrial, scientific, and medical (ISM) bands for tag-reader communication [8]. Considering the current demand in the food industry, it is important to record the history of changes in prices of all items inside the food chain. A conventional RFID tag can read a single parameter for processing, and when prices in the food chain are changed, all RFID tags must be redesigned or reconfigured. Redesign and manual reconfiguration of the tags requires significant time. In wireless sensor application research, chipless resonator tags for RFID are at the forefront of the conventional barcode replacement. The RFID tag is still larger in dimensions, which is a matter of concern for researchers. An RFID sensor tag comprises two separate antennas working as Tx and Rx, one transmission line, and resonators. The resonators have various shapes. It is currently very important to design an effective resonator that can be of smaller size, produce multiple resonances, and be reused conveniently, thus eliminating the need for more than one resonator in a tag.

In this paper, we have proposed a resonator structure based on substrate integrated waveguide (SIW) technology that can be easily incorporated into a chipless RFID tag. The resonator works at 16 different frequencies in a range of 2.45 to 3.05 GHz. It has four empty vertical channels; when a single channel is filled with liquid metal (LM), a new resonating frequency becomes available. Based on the four channels, this structure gives a combination of multiple LM-filled vertical channels which produce multiple frequencies. The number of resonant frequencies produced from the different combinations of four vertical channels is 16. This also makes it easier to switch frequencies, and particular information can be retrieved at any time by allocating one of the frequencies. It is obvious that electronic switching devices such as MEMS or varactor diodes are much faster and practical than liquid metal injection. However, the proposed resonator is useful for reusable wireless RFID applications. It does not have to be quickly switched. When we need to change the information, we just need to inject liquid metal, although it is manually changed. For instance, although the conventional RFID tag’s information cannot be modified once it is printed or fabricated, the proposed RFID tag’s information can be modified by injecting liquid metal after it is printed or fabricated. Therefore, the proposed RFID tag can be repeatedly reused without re-designing or manufacturing. When prices in the food chain increase or decrease, we do not need to redesign the tag; we can use the 15 available frequency choices by simply filling the empty channels of the resonator with the LM and reading new information from a new frequency. This makes the resonator very much reusable.

## 2. SIW Resonator Design

SIW structures are promising because they can take advantage of waveguides and printed circuit boards (PCB). SIW structures can be easily integrated into microwave and millimeter-wave circuits with simplicity, low radiation loss, small size, and easy fabrication [9]. The greatest benefit of SIW is that it enables us to manufacture the entire circuit in a planar configuration comprising of circuitry, four-sided waveguides, transitions, and antennas using a standard PCB or any other method. Furthermore, there is also the option to mount one or more chipsets on the same dielectric material; therefore, there is no need to change from components developed by different systems, thus reducing parasitic losses. System-on-substrate characterizes the perfect platform for developing high-performance, easy-to-manufacture, and less expensive millimeter-wave products [10]. An SIW structure is made up of two rows of conducting vias connecting the upper and lower planes and is filled with a dielectric material.

The proposed structure can modify its electrical size easily by introducing EGaIn LM-filled vertical channels. The challenge in using EGaIn material and possible solutions are addressed in the literature [11,12,13,14]. Previous researches using liquid metal in RF electronics are also available [15].

**Figure 2:**
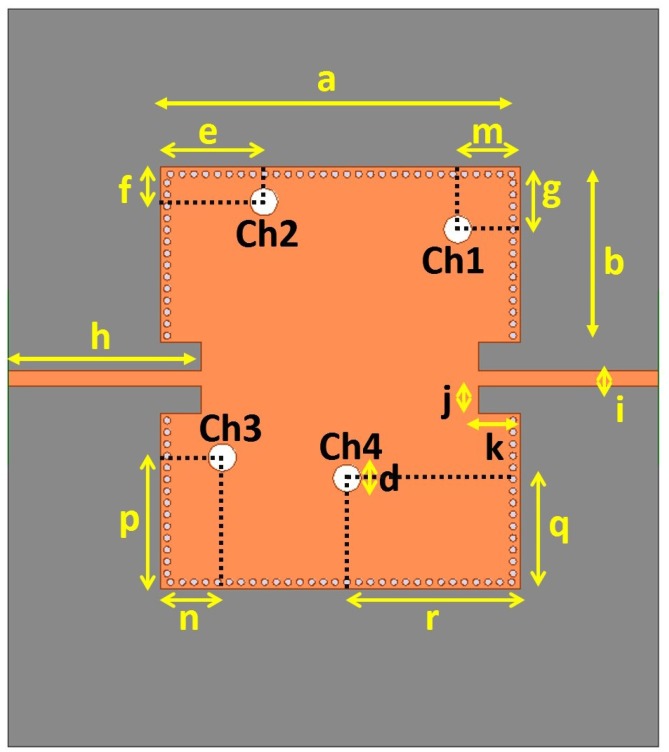
Layout of the proposed substrate integrated waveguide (SIW) resonator.

Figure 2 shows the layout of the proposed SIW resonator. The proposed structure is designed on a sheet of Rogers RT/Duroid 5870 substrate with a dielectric constant *ε_r_* of 2.33 and a thickness *h* of 0.79 mm. When the thickness of the SIW cavity *h* is much less than the width *W_SIW_* and length *L_SIW_*, the resonant frequency of the SIW cavity is determined by:

(1) $$f_{mn}=\frac{1}{2\pi \sqrt{\mu \epsilon }}\sqrt{{\left(\frac{m\pi }{W_{SIW}}\right)}^{2}+{\left(\frac{n\pi }{L_{SIW}}\right)}^{2}}$$

where *ϵ* and *μ* are the permittivity and permeability of the dielectric material of the SIW structure respectively, *m* and *n* are the mode numbers of the SIW cavity. Therefore, in order to achieve the resonant frequency of the cavity at 2.4 GHz, *L_SIW_* (=a) is 52 mm and *W_SIW_* (= 2 × (*i* + *j* + *b*)) is 61 mm.

This structure has four vertical channels as seen in Figure 2; they are labelled Ch1, Ch2, Ch3, and Ch4. Each channel has a diameter of 4 mm and is located at a different positions on the aperture of the SIW. The detailed design parameters for the proposed SIW resonator shown in Figure 2 are given in Table 1. The locations of the four vertical channels and their distances from the center of the SIW are kept different to avoid frequency overlap. Each channel is initially empty and then filled with LM to switch frequencies. Hence, the four channels facilitate 16 possible LM injection states, and all 16 sixteen possibilities have different resonant frequencies. For example, if we denote an empty fluidic channel as “0” and a filled fluidic channel as “1”, when four channels are empty, this structure resonates at 2.45 GHz (0000); when all four channels are injected with LM (1111), the resonant frequency is 3.05 GHz. In addition, there are 14 other possible LM injection combinations (states) with another 14 frequencies between 2.45 and 3.05 GHz. The 16 possible LM injection states are shown in Table 2, where a “0” denotes an empty state and a “1” denotes an LM-filled state.

**Table 1:** Detailed design parameters of proposed SIW resonator.

Parameter	Dimension (mm)	Parameter	Dimension (mm)
a	52	n	9
b	25.36	p	19
d	4	q	16
e	15	r	25
f	5	i	2.28
g	9	j	4
m	9	k	6

**Table 2:** 16 possible liquid metal injecting states of the proposed SIW resonator.

State	Channel 1	Channel 2	Channel 3	Channel 4
1	0	0	0	0
2	0	0	0	1
3	0	0	1	0
4	0	0	1	1
5	0	1	0	0
6	0	1	0	1
7	0	1	1	0
8	0	1	1	1
9	1	0	0	0
10	1	0	0	1
11	1	0	1	0
12	1	0	1	1
13	1	1	0	0
14	1	1	0	1
15	1	1	1	0
16	1	1	1	1

Each state shown in Table 2 has its own resonant frequency. The resonator can be easily reused 15 times in the RFID tag as a multiresonator, that fulfils the demand for multiple frequency usability in a single RFID wireless sensor system.

## 3. Simulation Results

A full-wave simulation is performed by an ANSYS high-frequency structure simulator (HFSS). In the HFSS material library, the EGaIn (LM) is generated by defining its bulk conductivity as (*σ* = 3.4 × 10^4^ Scm^−1^).

**Figure 3:**
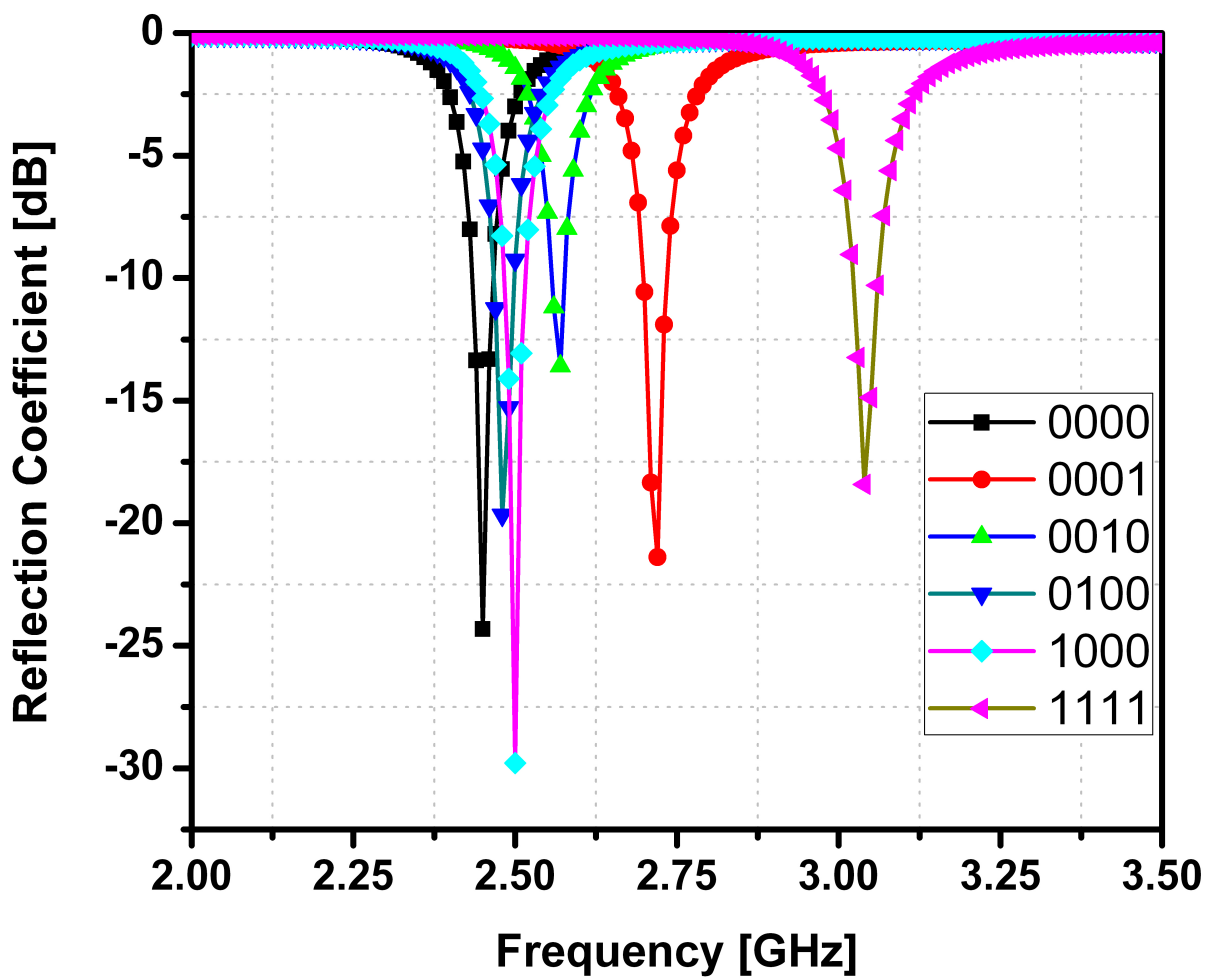
Simulated reflection coefficients of the proposed SIW resonator.

**Figure 4:**
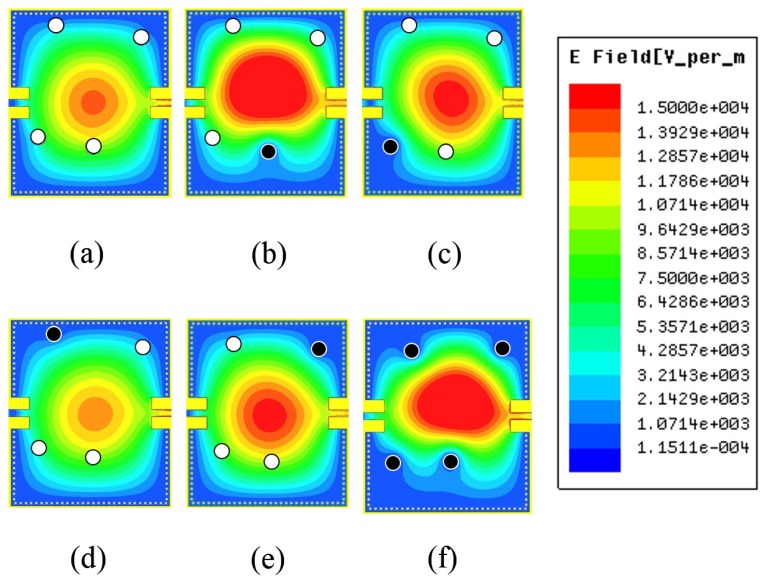
Magnitude of Electric Field Distributions at: (**a**) 0000; (**b**) 0001; (**c**) 0010; (**d**) 0100; (**e**) 1000 and (**f**) 1111.

Figure 3 shows the simulated reflection coefficients of the designed SIW resonator with six different LM injecting states: “0000”, “0001”, “0010”, “0100”, “1000”, and “1111”. As the vertical channel is filled with LM, a new resonant frequency is produced. There is coupling generated between the LM-filled vertical channels so that the LM-filled channels affect each other. However, it is important to generate the combination of resonant frequencies as many as possible from four channels. In the proposed work, four channels are asymmetrically located and all distance between them are different after optimization. Therefore, we can generate 16 combination of the resonant frequency from 4 channels. In Figure 3, when all four channels are empty, the resonant frequency is 2.45 GHz (black trace), and when all channels are LM filled, the resonant frequency is 3.05 GHz (pink trace). The positions of the vertical channels are selected to avoid overlapping of the resonant frequencies produced (achieving 16 distinct resonant frequencies). It was observed that when an LM-filled channel is close to the center of the SIW, maximum frequency switching is observed. This is because the magnitude of electric field distribution in the SIW is maximum towards the center. The magnitude of the e-field distribution in the SIW for six different LM states can be seen in Figure 4. The resonant frequency at (a) “0000” state is 2.45 GHz; at (b) “0001” is 2.71 GHz; at (c) “0010” is 2.56 GHz; at (d) “0100” is 2.48 GHz; at (e) “1000” is 2.5 GHz and at (f) “1111” is 3.05 GHz. It is demonstrated from the simulated results that there are 16 distinct frequencies in the range from 2.45 to 3.05 GHz. On the other hands, as shown in Figure 4, the magnitude of electric fields at each state is different. Therefore, input impedance and intrinsic Q factor of the resonator at each state are expected to be different. It results that the extinction ratio and coupling efficiency are changed at different resonant frequencies. However, it is more important to achieve different resonant frequencies at all states rather than constant extinction ratio and coupling efficiency because each information corresponds to the resonant frequency in RFID applications. Therefore, we elaborated on generating 16 resonant frequencies from 4 channels.

## 4. Experimental Demonstration

**Figure 5:**
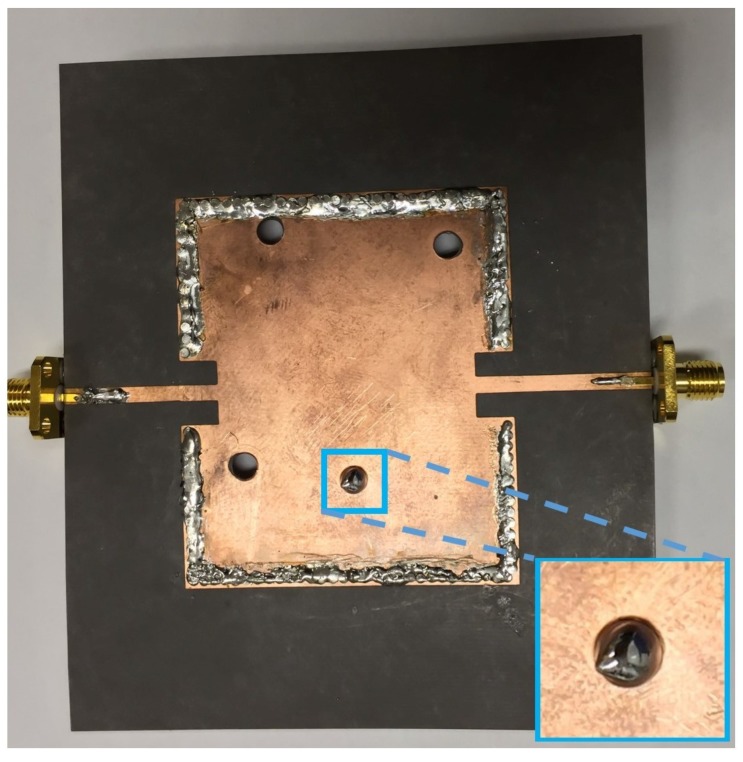
Photograph of the fabricated prototype at state 2 (0001). The magnified inset shows the liquid metal (LM)-filled channel after injecting eutectic gallium indium (EGaIn) into the channel 4.

**Figure 6:**
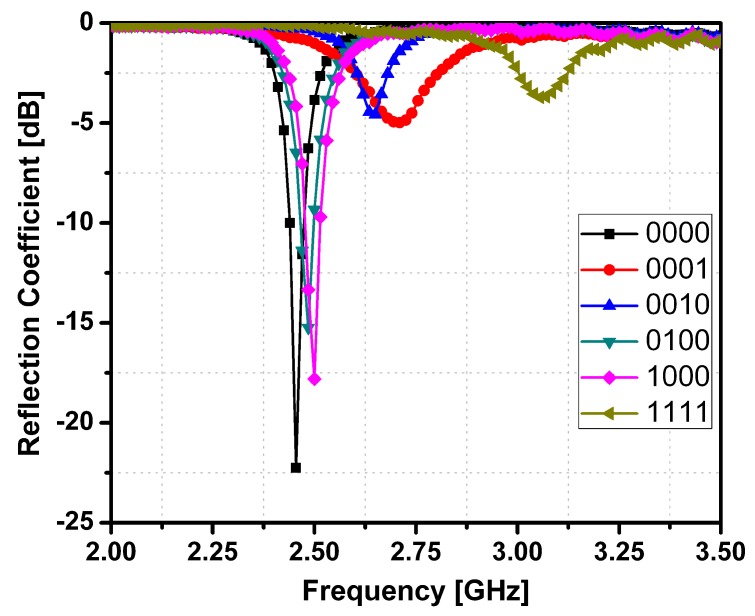
Measured Reflection Coefficients of Proposed SIW resonator.

Figure 5 shows the photograph of the fabricated prototype. The SIW cavity is build on Rogers RT/Duroid 5870 substrate with a dielectric constant *ε_r_* of 2.33 and a thickness *h* of 0.79 mm. The four channels are formed by drilling holes with 4 mm diameter. As an example, the state 2 is shown in Figure 5 where only channel 4 is filled with EGaIn and other three channels are empty. The magnified inset shows the LM-filled channel after injecting EGaIn into the channel 4. It undergoes the process of PCB etching, by first embossing the top SIW pattern on the substrate sheet using ultra violet light exposure system and then putting the sample into chemical etching liquid at 30 degrees centigrade. The sample is kept inside the etching liquid till the substrate material and the copper are completely separated. The four vertical channels with a diameter of exactly 4 mm were constructed using the drill machine. The bottom of the vertical channels is covered by copper tape to sustain the LM inside the channels. A normal sized syringe is used to inject LM into the vertical channels. The reflection coefficients of the proposed SIW resonator are measured using an HP 8510C network analyzer. The measured reflection coefficients of the six LM injecting states are shown in Figure 6. The reliability of the LM switching method is verified by injecting and extracting the LM repeatedly. The resonant frequencies of all 16 states are constant each time.

Table 3 gives a detailed comparison of the simulated measured resonant frequencies and Q-factors for all the 16 LM states of the proposed SIW resonator. The measured resonant frequencies show good agreement with the simulated resonant frequencies. On the other hands, the measured Q-factors are lower than the simulated Q-factors due to parasitic losses from fabrication and measurement.

The tuning ratio (TR) of the proposed SIW resonator is compared with TR of the other state-of-the-art reconfigurable SIW resonators in Table 4. TR is defined as

(2) $$TR\left[\%\right]=\frac{f_{H}-f_{L}}{f_{c}}\times 100$$

where *f_c_* is the average of the highest frequency *f_H_* and the lowest frequency *f_L_*.

**Table 3:** Comparison of simulated and measured resonant frequencies and Q-factors of 16 liquid metal injecting states of the proposed SIW resonator.

State	Channel 1	Channel 2	Channel 3	Channel 4	Frequency (GHz)		Q-Factor	
					Sim.	Meas.	Sim.	Meas.
1	0	0	0	0	2.45	2.45	24.5	24.22
2	0	0	0	1	2.71	2.69	24.72	22.52
3	0	0	1	0	2.56	2.56	28.55	25.4
4	0	0	1	1	2.85	2.85	35.62	22.75
5	0	1	0	0	2.48	2.48	24.8	21.4
6	0	1	0	1	2.77	2.77	23	20.73
7	0	1	1	0	2.69	2.69	33.6	20.31
8	0	1	1	1	2.92	2.92	32.33	19.91
9	1	0	0	0	2.50	2.50	25	19.5
10	1	0	0	1	2.97	2.95	27.09	19.43
11	1	0	1	0	2.60	2.59	29	19.31
12	1	0	1	1	2.90	2.90	29	19.24
13	1	1	0	0	2.53	2.53	25.3	19.18
14	1	1	0	1	2.86	2.85	23.91	19.15
15	1	1	1	0	2.75	2.74	27.6	19.1
16	1	1	1	1	3.05	3.05	25.41	19.08

**Table 4:** Performance comparison of the proposed resonator with other reconfigurable SIW resonators.

	This Study	[9]	[16]	[17]	[18]
*f_L_*∼*f_H_* (GHz)	2.45∼3.05	2.28∼2.50	2.40∼2.50	2.60∼3.10	1.55∼2.0
Technology	LM	Varactor	Varactor	Varactor	PIN diodes
TR (%)	22	9.2	4	17.47	25.35
Size (*λ*_0_ × *λ*_0_)	0.53 × 0.62	0.21 × 0.21	0.22 × 0.11	0.27 × 0.27	0.22 × 0.42
Transmission line	SIW	QMSIW	EMSIW	SIW	SIW

QMSIW = Quarter-Mode SIW; EMSIW = Eighth-Mode SIW.

## 5. Conclusions

A reusable EGaIn-injected resonator for wireless sensor applications was proposed using SIW technology. The capability to reuse multiple frequencies was achieved by injecting EGaIn LM alloy into the four vertical fluidic channels. By injecting EGaIn (LM), it was successfully demonstrated that there are 16 different resonant frequencies from 2.45 to 3.05 GHz. The switch states of the EGaIn could be manually controlled in real time by integrating the micropump. The manual switching method using EGaIn was slower than electronic switching method. However, the proposed SIW resonator can be effectively used in the food chain industry, where multiresonators in RFID tags are required for frequency reusability, which helps acquired information to be read without redesigning the tag. In addition, the proposed SIW resonator is also suitable for future substrate integrated circuits or systems-on-substrate in wireless sensors, which require frequency reuse capability as well as multiband wireless sensor applications.
